# Comparative Review of Marshall and Superpave Mix Designs: Enhancing Asphalt Performance with Polymers

**DOI:** 10.3390/ma18184273

**Published:** 2025-09-12

**Authors:** Gulzar Hussain Jatoi, Giuseppe Loprencipe, Laura Moretti

**Affiliations:** 1 Department of Civil, Building and Environmental Engineering, Sapienza University of Rome, Via Eudossiana 18, 00184 Rome, Italy; gulzarhussain.jatoi@uniroma1.it (G.H.J.); laura.moretti@uniroma1.it (L.M.); 2 Department of Civil Engineering, Aror University of Art, Architecture, Design and Heritage, RCW Rohri Bypass, Sukkur 65170, Sindh, Pakistan

**Keywords:** Marshall mix design, Superpave mix design, polymer-modified bitumen, rejuvenators

## Abstract

The durability of asphalt pavements is crucial for sustainable road infrastructures. This systematic review compares the Marshall and Superpave asphalt mix design protocols, with a particular focus on the integration of polymer-modified bitumen (PMB) and rejuvenators. Although the Marshall method remains widely used for its simplicity and cost-efficiency, its empirical basis limits its effectiveness to meet modern pavement performance demands. In contrast, the Superpave system offers improved resistance to rutting, longer fatigue life, and better mitigation of moisture damage. The review traces the evolution of asphalt mix design, identifies current challenges, and emphasizes the need for transitioning toward performance-based frameworks. Special attention is given to the incorporation of polymers such as Styrene–Butadiene–Styrene (SBS), Styrene–Butadiene–Rubber (SBR), and Polyethylene (PE), which significantly enhance the mechanical properties of asphalt mixtures. The role of rejuvenators in restoring aged binders and enabling pavement recycling is also examined. Finally, the manuscript provides strategic recommendations for adopting Superpave to enhance pavement durability and reduce lifecycle maintenance costs. Overall, this comprehensive review advances knowledge on asphalt mix design, fostering innovation and sustainability while promoting long-term resilience in road pavement infrastructures.

## 1. Overview of Asphalt Mix Design

As road infrastructure faces increasing traffic volumes and higher service demands, performance-based materials have become essential in addressing road durability challenges [1]. The design of asphalt mixtures plays a pivotal role in long-lasting and high-performing pavements [2]. The Marshall mix design remains widely used due to its simplicity, low equipment requirements, and cost-effectiveness [3]. However, despite these advantages, the Marshall method is inherently empirical, relying primarily on parameters such as stability and flow, which limit its ability to predict long-term field performance under varying climatic conditions and traffic load [3]. To address these limitations, the Strategic Highway Research Program introduced the Superpave (Superior Performing Asphalt Pavements) mix design approach. Superpave integrates mechanistic–empirical principles, incorporating binder performance grade (PG), aggregate angularity, and volumetric properties, while simulating field compaction more accurately through the gyratory compactor. This results in better alignment between laboratory predictions and field performance [4,5]. The Superpave system allows engineers to tailor mixtures to specific climate and traffic conditions, thereby offering enhanced resistance to rutting, fatigue, and moisture susceptibility [3,5]. The transition toward performance-oriented design is particularly critical in rapidly urbanizing regions with escalating traffic demand. For instance, studies in Pakistan have demonstrated that the widespread use of paving-grade 60/70 binders fails to meet performance requirements, reducing pavement lifespan and increasing maintenance works [3,5]. Farooq et al. [5] reported that Superpave mixtures had a higher indirect tensile strength (ITS) and resilient modulus (MR) than Marshall mixtures, indicating improved performance under traffic-induced stresses. Similarly, Zumrawi and Edrees [3] found that conventional mixes provided poorer rutting and thermal crack resistance compared to Superpave mixes in hot climate regions [3]. In this context, polymer-modified bitumens (PMBs) containing SBS or SBR have been shown to effectively improve fatigue life and resistance to permanent deformation [6]. The integration of performance-based approaches further facilitates material compatibility with local climate zones while enabling the utilization of recycled materials and sustainable practices [7].

**Figure 1:**
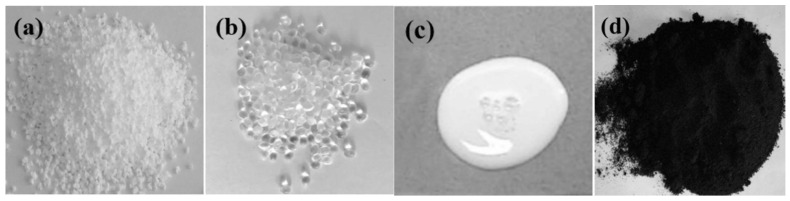
Polymers used for the modification: (**a**) SBS, (**b**) EVA, (**c**) SBR, and (**d**) rubber [9].

Bitumen, the most expensive component in asphalt mixtures, plays a pivotal role in determining pavement performance, particularly under extreme climatic conditions and heavy traffic demands [8]. Polymer modification has emerged as a critical advancement for enhancing binder properties. This process involves blending polymers with the base binder to improve viscoelastic behavior [9]. In recent years, PMBs, especially those modified with Styrene–Butadiene–Styrene (SBS), Styrene–Butadiene Rubber (SBR) and rubber, and plastomers including Polyethylene (PE), Polypropylene (PP) and Ethylene-Vinyl Acetate (EVA), have further demonstrated improvements in the mechanical performance of asphalt mixtures (Figure 1).

PMB has significantly enhanced rutting resistance, thermal stability, fatigue life, crack resistance, elasticity, and aging performance [9,10]. PMBs are particularly effective in high-temperature environments where unmodified bitumen is prone to excessive deformation. The interaction between bitumen and polymers can be either physical or chemical, depending on the polymer type. For instance, SBS absorbs maltenes from the binder, swelling up to nine times its original volume and forming a gel-like network that greatly improves elasticity and cohesion [11]. In addition, the incorporation of rejuvenators plays a critical role in restoring the rheological properties of aged binders, thereby enabling the effective utilization of reclaimed asphalt pavement (RAP) and supporting the circular economy within the asphalt industry [7]. Their effectiveness has been confirmed by the observed reduction in global virgin bitumen consumption between 2021 and 2024 [9,12]. By facilitating RAP recycling, rejuvenators contribute to the production of asphalt mixtures with improved durability and mechanical performance [13]. This not only strengthens recycling loops within the asphalt sector but also saves natural resources through reduced reliance on virgin bitumen.

The objective of review is to investigate the influences of polymer-modified asphalt (PMA) on the performance of the asphalt mixtures with respect to fatigue, rutting, and cracking resistance, and moisture susceptibility. The evaluation compares the performances achieved through the Marshall Mix Design and Superpave Mix Design, with the aim of determining whether recent changes in mix design have significantly influenced the polymers utilization. Furthermore, the review discusses how these advancements in mix design affect the long-term durability and service life of asphalt pavements, with particular emphasis on the effectiveness of rejuvenators in enhancing recycled asphalt content and supporting sustainable pavement practices.

## 2. Types of Polymers

Elastomers are thermoplastic polymers characterized by their rubber-like elasticity. Common examples include SBS, SBR, styrene–isoprene–styrene (SIS), and styrene–ethylene–butylene–styrene (SEBS) [14,15]. These polymers are widely applied in asphalt modification because they enhance elastic recovery while improving fatigue and crack resistance. In particular, SBS and SBR have demonstrated significant improvements in elastic recovery, fatigue resistance, and crack mitigation, especially under low-temperature conditions [9,10,16,17]. When mixed with bitumen, elastomers swell and form a continuous elastic network that substantially improves the rheological properties of bitumen under varying stress and temperature conditions [12,18]. This swelling process undergoes visco-rubber/viscoelastic phase inversion, producing a polymer-rich phase that allows viscoelastic behavior to dominate the asphalt matrix [12]. The key benefits of elastomer modification include enhanced elastic recovery, improved resistance to fatigue and cracking, superior low-temperature performance, and increased resistance to aging and oxidation [18,19,20].

According to Table 1 [9], even small amounts of elastomer can significantly reduce asphalt penetration while raising the softening point of the binder. For instance, the addition of 5% SBS by weight can increase the softening point to as high as 95 °C [21]. Moreover, elastomer-modified binders demonstrate elastic recovery exceeding 50% at 5 °C [3,21]. SBS elastomers are also employed to enhance elastic recovery and rutting resistance in mixtures containing waste motor oils, which help reduce binder viscosity and lower mixing and compaction temperatures [22].

Plastomers are thermoplastic polymers that behave like plastics and are primarily used to enhance the stiffness, rigidity, and rutting resistance of asphalt binders. However, their incorporation often reduces binder flexibility at low temperatures. Common plastomers used in asphalt modification include polyethylene (PE), polypropylene (PP), ethylene-vinyl acetate (EVA), ethylene/methacrylic acid copolymer (EMA), and ethylene-butyl acrylate copolymer (EBA) [9,10]. Among these, EVA is one of the most widely applied plastomers, known for improving the rigidity, stiffness, and rutting resistance of bitumen binders. Nonetheless, EVA modification may also decrease the flexibility of bitumen under low-temperature conditions. According to Table 2, the performance of plastomer-modified asphalt varies depending on the type and dosage of plastomer used. For instance, asphalt modified with a small amount of EVA undergoes significant crystallization, yielding properties comparable to low-density polyethylene (LDPE). At moderate concentrations, EVA enhances binder compatibility and storage stability [23]. However, when the EVA content exceeds 4 wt%, the degree of crystallization decreases, which may lower resistance to low-temperature cracking [23,24,25]. Moreover, excessively high EVA dosages can result in poor compatibility with bitumen, thereby diminishing the overall performance benefits [25]. Although plastomers are generally cost-effective, they significantly improve rutting resistance and high-temperature performance. Furthermore, certain plastomers (e.g., EVA) provide better storage stability due to their polar functional groups, which enhance interaction with the bitumen matrix [17,25,26].

Furthermore, combining polymer modification with sustainable materials (e.g., graphene additives, recycled plastics, and rejuvenators) offers a dual benefit: improved pavement performance [27] and reduced environmental impact [28,29,30]. When incorporated through either dry or wet processes, these modifiers enhance binder stiffness, extend fatigue life, and increase recyclability.

Table 3 compares the characteristics of elastomers and plastomers [10,26,31,32,33,34].

**Figure 2:**
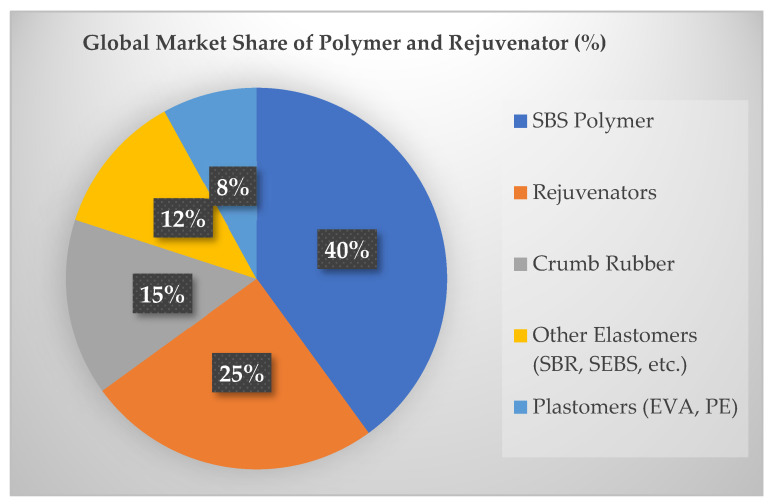
Global market share of polymer and rejuvenator.

Figure 2 shows the global percentage of polymer types used in asphalt mixtures, along with the corresponding market share of rejuvenators [12,35,36].

Modifying bitumen with plastomers and elastomers plays a crucial role in enhancing the performance and durability of asphalt mixtures [37]. PMA can better withstand harsh environmental conditions [23] and heavy traffic loads by improving mechanical properties, making it a highly effective solution [19,38].

Similarly, the incorporation of recycled materials into asphalt mixtures improves sustainability and reduces the environmental impact of road construction [39]. Recent advancements include the use of bio-polymers such as poly β-hydroxybutyrate-co-β-hydroxy valerate (PHBV), which serve as “green” alternatives to petroleum-based modifiers [40]. Furthermore, the assessment of microplastic (MP) release from recycled polymer-modified asphalt has become increasingly important for evaluating potential environmental impacts [41].

## 3. Methodology

### 3.1. Design

This review adopts a systematic literature review (SLR) approach to collect, analyze, and synthesize data [42] on the performance of PMAs and recent developments in asphalt mix design. The primary focus is on comparing the Marshall and Superpave mix design methods while examining the role of SBS and SBR in improving asphalt performance. Additionally, the review emphasizes sustainability by exploring the integration of recycled plastics into asphalt mixtures. Overall, the study seeks to summarize and evaluate current knowledge, highlighting the transition from Marshall to Superpave mix design and assessing the influence of different polymers on asphalt mixture performance.

The methodology involves identifying research gaps and emerging trends, as well as comparing outcomes across various PMAs. The review critically evaluates previous scientific studies to determine their relevance for asphalt design, with a particular focus on the effects of polymer compounds on fatigue and rutting resistance. Following the PRISMA (Preferred Reporting Items for Systematic Reviews and Meta-Analyses) guidelines, only scientifically rigorous and thematically relevant studies were included [43]. This structured approach enhances validity, reduces potential biases, and strengthens the overall quality of the findings. It enables a detailed comparison of methods, practices, and results the impact of PMA’s on pavement durability [44].

The review process was guided by predefined goals and research questions, aligned with PRISMA criteria. Initially, keywords were refined to capture the most relevant scientific papers. Subsequently, filters were applied to narrow the scope of literature. After careful screening and selection, the publications were analyzed, categorized, and discussed systematically to ensure comprehensive coverage of the topic.

### 3.2. Study Question

Traditionally, asphalt mixtures were designed using the conventional Marshall design method, which was since been largely replaced by the performance-based Superpave mix design approach. Unlike the Marshall method, Superpave better replicates actual field conditions, thereby addressing common pavement failures. In addition, various polymers have been incorporated into asphalt binders to further improve performance. Polymer modification has been shown to significantly enhance rutting resistance, fatigue resistance, and moisture susceptibility.

This study focuses on comparing the Marshall and Superpave approaches in the scientific literature. Guided by the development of a suitable research question (Table 4), a systematic literature review was conducted to ensure a comprehensive and structured evaluation of existing studies.

This review ensured a comprehensive and systematic collection of relevant information by searching major scientific databases, including Web of Science (WoS), Scopus, and ScienceDirect. These platforms provided access to high-quality publications related to PMA mixtures. Research articles were retrieved using optimized search strings (Table 5), with several combinations of search phrases applied in each database to align with the questions in Table 4. Keywords such as polymer-modified asphalt, asphalt mixtures, Marshall mix, and Superpave mix were specifically employed to capture relevant literature. Using this approach, a total of 1115 publications were retrieved, including articles, review papers, and conference proceedings.

### 3.3. Data Filtering

**Figure 3:**
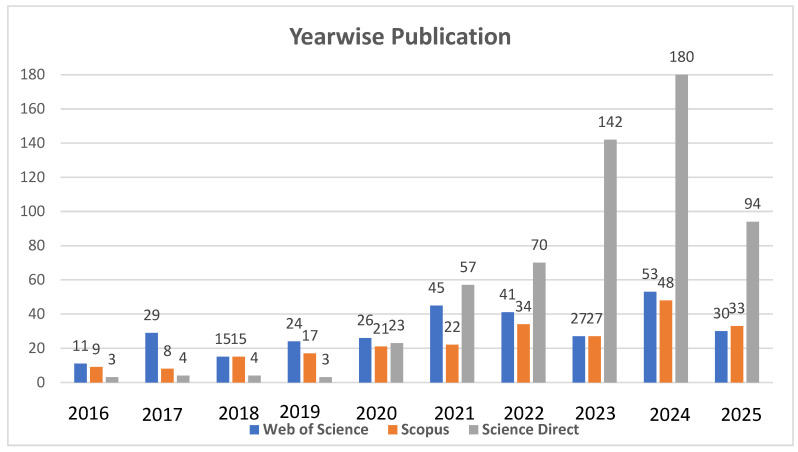
Year-wise publication in different databases.

Several filters were applied during the research process to ensure the quality, consistency, and relevance of the selected studies. A structured methodology was followed for data extraction, organization, and analysis [44]. After carefully screening, studies were documented, justified, and extracted in alignment with the research objectives and questions. Key characteristics considered included PMB, RAP content, design methods, and mechanical properties of asphalt mixtures. A consensus process was used to validate the collected data, allowing for comparisons findings across various studies to identify patterns, differences, and comparisons across studies to identify patters, similarities, and differences. Only studies meeting the eligibility criteria were included, and all information was systematically organized (Figure 3). The publication trend shows that 2016 recorded the fewest relevant publications, with only 23 across all three databases. In subsequent years, the number of publications steadily increased, peaking in 2024 with 281 publications. Notably, since 2021 there has been a marked surge in outputs from ScienceDirect, which accounted for the highest number in 2024, with 180 publications directly related to this study.

The VOS Viewer tool (version 1.6.20) identified four keyword clusters in the selected studies:

1 The green cluster focuses on the performance of asphalt mixtures, particularly the behavior of reclaimed asphalt under various conditions (e.g., moisture, stress, temperature, and aging). It addresses critical issues such as rutting, fatigue, cracking, and durability.
2 The red cluster concentrates on the mechanical properties of asphalt mixtures, especially when recycled materials are incorporated. Key aspects include durability, toughness, strength, and microstructure, often examined within a broader sustainability framework.
3 The blue cluster examines the rheological and material properties of asphalt binders and mixtures, especially when modified with polymers, rejuvenators, additives, and recycled materials.
4 The yellow cluster investigates rejuvenators and binders, focusing on the use of soft bitumen and rejuvenating agents to restore aged asphalt binders in reclaimed mixtures. This cluster emphasizes improving flexibility and ensuring compatibility with high percentages of RAP.

**Figure 4:**
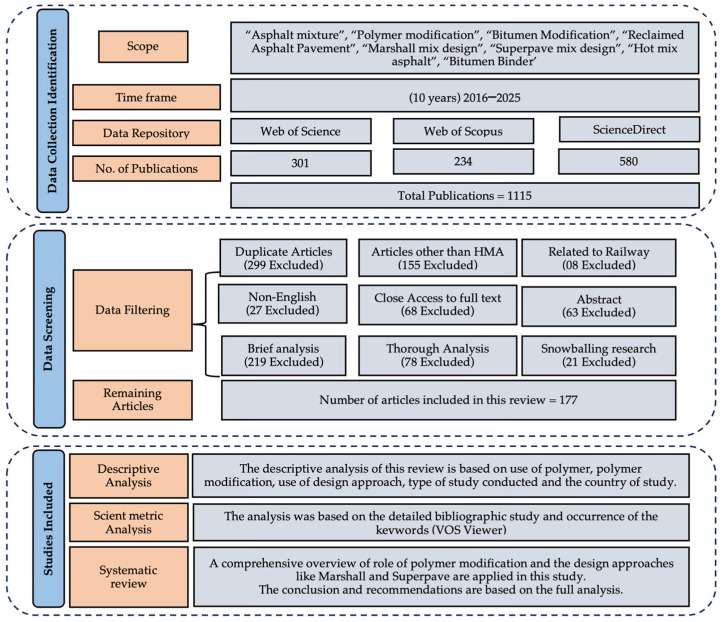
PRISMA study flow chart.

Figure 4 presents the PRISMA diagram illustrating the systematic review search and filtering process. The screening involved several steps, including the removal of duplicate records, exclusion of closed-access articles, elimination of studies related to railway ballast or track layering, and filtering based on titles and abstracts. From the initial 1115 publications identified, 177 met the eligibility criteria and were included in this review. Among these, 15 significant studies are summarized in Table 6.

## 4. Discussion

A comprehensive systematic review of scientific studies was conducted to evaluate, identify, and select research specifically addressing polymer modification of bitumen and asphalt, comparisons between Marshall and Superpave mixtures, and the performance of PMAs. This review also examined the effects of elastomers and plastomers on the mechanical properties of asphalt mixtures within the framework of various design methods. To ensure quality and relevance, only studies meeting strict selection criteria were included. The final set of studies covers a broad range of research objectives, methodologies, and approaches. Many of them directly compare the Marshall and Superpave mix design methods [3,4,5], highlighting both approaches as efficient and suitable under challenging conditions. Additionally, numerous studies investigate the role of polymers and rejuvenators in asphalt mixtures to mitigate pavement failures [9,10,45,52,53]. Findings on Marshall versus Superpave mix designs are consistent with those from similar research [3,4,5,51,54,55]. Other studies emphasize polymer modification and the incorporation of rejuvenators during asphalt mixing, showing how these measures can reduce road distress. Comparisons between Marshall and Superpave mixtures reveal that Superpave tends to outperform Marshall under repeated loading.

In this section, a detailed analysis of selected elastomers (e.g., SBS and SBR), plastomers (e.g., PP and PE), and rejuvenators is proposed. Their performance is compared within Marshall and Superpave mix design framework. The discussion also explores how polymer modification influences the mechanical behavior of asphalt mixtures and evaluates its effectiveness in high-traffic and high-temperature conditions.

### 4.1. Elastomer Modified Asphalt in Comparison of Marshall Versus Superpave Method

#### 4.1.1. Styrene Butadiene Styrene (SBS)

SBS is a thermoplastic elastomer widely used to enhance asphalt performance. Incorporation of SBS improves the bitumen elasticity, fatigue resistance, and resistance to temperature susceptibility [56,57,58]. Its higher stiffness and elasticity also reduce permanent deformation [59]. The dynamic shear resistance (DSR) tests demonstrate that SBS-modified bitumen exhibits an increased complex modulus (G*) and a reduced phase angle (δ), resulting in improved rutting resistance [33]. Due to its elastic nature, SBS improves fatigue resistance under repeated cyclic loading [6,59,60]. Moreover, SBS-modified binders show slower oxidative aging, confirmed by laboratory tests such as DSR and FTIR aging indices [6,61].

In Marshall stability design, the inclusion of SBS increases stability, thereby improving load-bearing capacity and rutting resistance. Flow values remain within the optimum range, demonstrating a balance between flexibility and stiffness [6]. Additionally, SBS-modified mixtures exhibit superior resistance to stripping and moisture due to stronger adhesion between aggregate and binder, as indicated by surface free energy and pull-off tests [62]. However, the empirical Marshall method cannot fully capture the improvements in fatigue and rutting resistance provided by SBS; these properties are better evaluated using the Superpave method [63].

**Figure 5:**
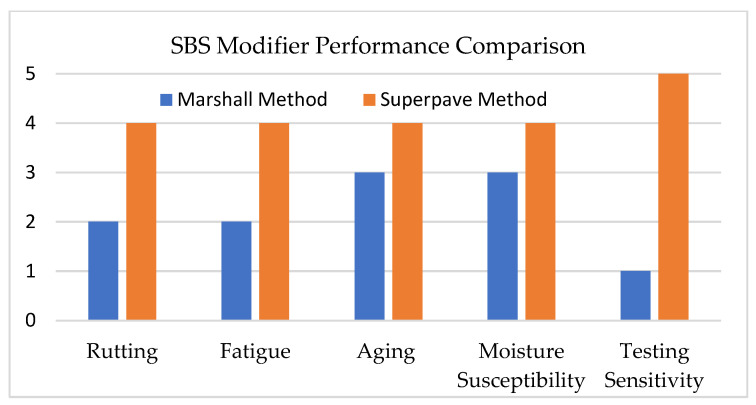
SBS Polymer Performance Comparison.

Figure 5 shows the performance of SBS as an asphalt modifier under both methods [6,9,11,33,37,45,52,53,60,61,62,64,65,66,67,68]. Each score was derived by interpreting the test capabilities and performance outcomes of both methods, according to a qualitative scale from 0 to 5, where 0 means very poor, 1 poor, 2 fair, 3 good, 4 very good, and 5 excellent.

#### 4.1.2. Styrene Butadiene Resin (SBR)

SBR is an important asphalt modifier that improves key performance parameters in both mix design methods, though its benefits are more accurately assessed under the Superpave framework [69]. In Superpave testing, SBR enhances resistance to rutting, particularly in mixtures containing RAP. It also improves fatigue resistance through greater elastic recovery and increased durability under repeated loading [37,53,70,71]. Furthermore, SBR enhances moisture resistance, as demonstrated by higher tensile strength ratio (TSR) values and improved indirect tensile strength (ITS) [9,70]. However, SBR-modified binders are more susceptible to oxidative aging. Superpave aging protocols, such as Rolling thin film oven test and Pressure Aging Vessel, provide specific insights into this behavior [9,26,72]. While the Marshall method also reflects improvements in stability and moisture resistance, its empirical nature does not capture the mechanistic advantages of SBR [73].

**Figure 6:**
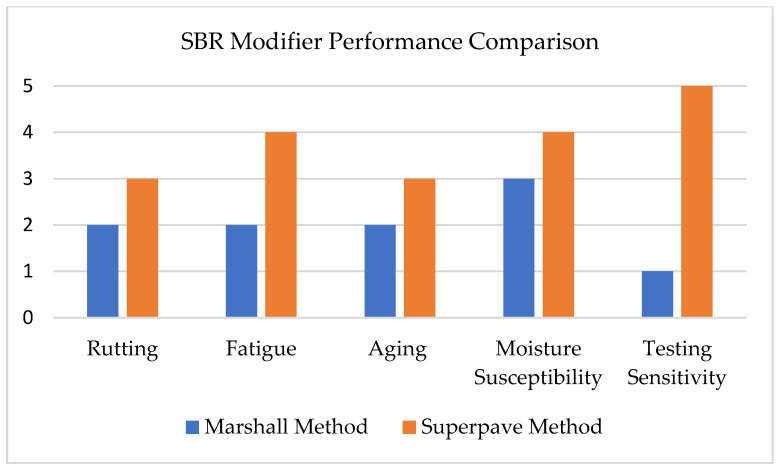
SBR Polymer Performance Comparison.

Figure 6 shows the performance of SBR as an asphalt modifier in both methods [9,10,11,70,71,73,74,75,76,77,78]. The representation criterium complies with that in Figure 5.

### 4.2. Plastomers Modified Asphalt in Comparison of Marshall Versus Superpave Method

#### 4.2.1. Polyethylene (PE)

Polyethylene (PE), a plastomer often used in recycled form, is a widely applied modifier due to its ability to improve high-temperature performance, rutting resistance, and stiffness. Positive effects of PE-modified binders have been documented in both methods [79,80,81]. According to Superpave protocols, PE increases PG of binders, particularly in hot climates, by significantly reducing non-recoverable creep compliance and enhancing the asphalt stiffness [82,83,84]. In the Marshall mix design, PE increases Marshall stability, reduces flow values, and raises the softening point. However, excessive PE content can lead to brittleness and reduced ductility [85,86]. Laboratory studies show that adding 5–7% PE can increase rutting resistance by up to 33% and resilient modulus by 55% compared to virgin bitumen [85].

Challenges with PE modification arise from its nonpopular nature and crystalline structure, which lead to compatibility issues, storage instability, and phase separation. To address these limitations, strategies such as co-blending PE with elastomers (e.g., SBS), chemical pretreatment, or the addition of nanoclay and pyrolytic wax have been explored to improve dispersion and reduce brittleness [80,87].

Overall, PE enhances high-temperature and deformation performance in both design methods. However, its limitations demand supplementary treatments or hybrid modification strategies.

#### 4.2.2. Polypropylene (PP)

Polypropylene, a hydrocarbon thermoplastic polymer, is also employed as an asphalt modifier to improve mixture performance. Recently, the use of post-consumer PP has been studied as a sustainable approach [88,89]. PP enhances high-temperature stability, rutting resistance, and aging resistance, especially in hot regions and under heavy traffic [75,89,90,91].

In Superpave mix design, PP-modified binders exhibit superior rheological performance, with increased G* and reduced δ, leading to better rutting resistance [92]. The results from DSR and Multiple Stress Creep and Recovery tests confirm that PP fibers significantly reduce non-recoverable creep compliance and improve percent recovery, indicating better elastic recovery and deformation resistance. PP also improves oxidative aging resistance and moisture tolerance [9,89,93].

Within the Marshall framework, PP increases stability, stiffness modulus, and TSR. Incorporation of 2–6% PP raises optimum bitumen content, reduces moisture susceptibility, and increases softening point and stiffness while reducing penetration values. However, higher PP contents can reduce ductility, increase viscosity, and cause phase separation, thereby reducing processability [9,85,93,94,95]. Pretreatment techniques, such as pyrolysis to produce polypropylene wax (PPW), have been proposed to improve workability, minimize segregation, and enhance bonding between binder and aggregates [96]. The inclusion of PPW in both methods enhances rutting resistance and facilitates the use of warm mix asphalt technologies, reducing energy consumption during production [80,89,93].

### 4.3. Rejuvenators

Rejuvenators play a critical role in sustainable asphalt pavement practices by restoring the properties of RAP. Their primary function is to reverse binder oxidation, thereby improving workability, enhancing cracking resistance, and reducing stiffness [28,97,98].

**Figure 7:**
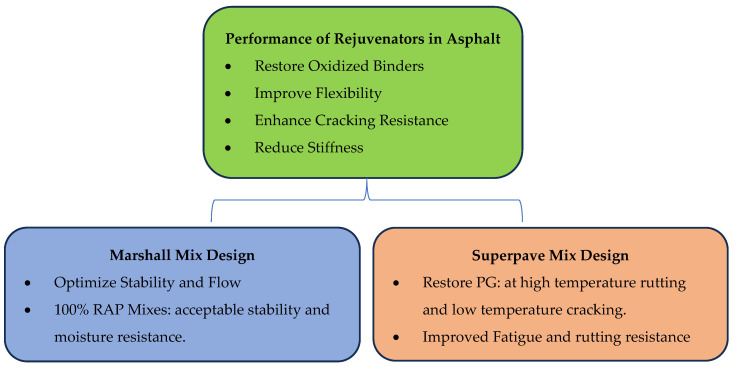
Performance of Rejuvenators.

In Superpave mix design, rejuvenators restore binder PG properties, ensuring compliance with high-temperature rutting and low-temperature cracking requirements. Vijayan et al. [7] demonstrated that a plant-based rejuvenator, combined with RAP and plastic-modified asphalt via the dry process, maintained satisfactory mechanical performance even after two recycling times. Improvements were observed in rutting resistance, moisture sensitivity, and fatigue properties [7,99,100,101]. In Marshall mix design, rejuvenators enhance stability and flow values by softening aged binders [99]. Full-scale mixtures containing up to 100% RAP, when adequately treated with rejuvenators, achieved acceptable stability and moisture resistance. Results based on traditional Marshall criteria—such as air voids and voids in mineral aggregate (VMA)—support their suitability [99,102,103]. According to [28], the synergistic effect of rejuvenators with the recycled polymers gave satisfactory properties even after multiple recycling cycles. Figure 7 summarizes the performance of rejuvenators in both mix design methods.

## 5. Conclusions

This review compared two methods of asphalt design (i.e., Marshall and Superpave) incorporating polymer modification and rejuvenators. The findings confirm that the Marshall method is an empirical widely adopted approach that is not performance-based and cannot predict long-term durability under high traffic loads and harsh climatic conditions. In contrast, the Superpave method provides a mechanistic–empirical design that enhances viscoelastic properties and resistance to moisture damage under varying traffic and climate conditions. The incorporation of polymers such as SBS, SBR, PP, and PE improves elasticity, stiffness, resistance to rutting, fatigue, and aging, while PMB offers an affordable option to enhance road performance. The addition of rejuvenators allows recycling RAP and plastic waste, supporting circular economy practices, restoring the rheological properties of aged binders, and contributing to sustainable development.

This review encourages further investigation into cost-effective solutions for local polymer modification, durability monitoring, and bridging the gap between sustainable asphalt practices and economy feasibility. It also highlights key future research directions, emphasizing the need to shift toward mechanistic–empirical design approaches. Future studies should also prioritize the selection of polymers based on local conditions, with a focus on economical modifiers (e.g., recycled plastics), and evaluate their performance through Marshall and Superpave methods. Additionally, long-term monitoring is crucial to assess the durability and efficiency of PMA, with particular emphasis on resistance to viscoelastic property degradation, moisture susceptibility, and overall performance in real-world conditions.

## Figures and Tables

**Table 1:** Summary of 150/220 grade asphalt characteristics through elastomer modification [9].

Asphalt Binder	160/220 Grade Asphalt (Control)	SBS (5% by Weight)	SEBS (3% by Weight)	SBR (5% by Weight)	Polybutadiene Rubber (1.5% by Weight)
Penetration @ 25 °C (dmm)	160–220	70–110	60–100	100–130	106
Softening point (°C)	35–43	75–95	65–85	56	48
Elastic recovery, @ 5 °C (%)	N/d	>50	>50	>70	>80
Fraass breaking point (°C)	−15	−20	−18	−15	−30

**Table 2:** Summary of the effect of plastic on asphalt and asphalt mixtures [9].

Polymers	70/100 Grade Asphalt (Control)	EVA 18/150 (5% by Weight)	EVA 30/45 (5% by Weight)	LDPE (4% by Weight)
Penetration at 25 °C (dmm)	70–100	38–48	55	47
softening point (°C)	43–51	58–68	57	53
Elastic Recovery, @ 5 °C (%)	20	50	50	82
Frass breaking point (°C)	−10	−18	−18	−3

**Table 3:** Characteristics of Elastomers and Plastomers.

Characteristics	Elastomers	Plastomers
Nature	Thermoplastic elastomers behave like rubber	Thermoplastics (behave like plastic)
Main Types Used	SBS, SBR, SEBS, SIS	PE, EVA, PP, EBA
Cost	Higher cost, but effective in small doses	Lower-cost, recycled plastics are widely available
Compatibility with Asphalt	Excellent compatibility forms a network in the binder	Moderate to poor requires compatibilizers
Elastic Recovery	Excellent elastic recovery	Limited elastic behavior
Rutting Resistance	Very high especially for SBS mixtures	High at higher temperatures especially with EVA
Low-Temperature Performance	Provides excellent flexibility and crack resistance	Poor brittle at low temps
Sustainability	Some options use reclaimed rubber (SBR)	PE, PP sourced from waste
Challenges	Oxidation of butadiene (SBR)	Crystallization, storage stability, and low elasticity

**Table 4:** Study questions.

Question 1	How PMB improves the performance of the mixture.
Question 2	Have the recent changes in approach to mix design led to any substantial differences in results between Marshall and Superpave using PMA?

**Table 5:** Repository and Search String.

ScienceDirect	Scopus		Web of Science
asphalt mixtures OR Polymer modified asphalt AND Rejuvenators OR recycled aggregate OR Marshall OR Superpave	TITLE-ABS-KEY (asphalt mixtures OR Polymer modified asphalt AND Rejuvenators OR recycled aggregate OR Marshall OR Superpave) AND PUBYEAR > 2015 AND PUBYEAR < 2026 AND (LIMIT-TO (DOCTYPE, “ar”) OR LIMIT-TO (DOCTYPE, “re”)) AND (LIMIT-TO (LANGUAGE, “English”))		“(TS = ((“asphaltic mixture” OR asphalt OR bitumen OR “asphalt concrete” OR pavement) AND (polymer OR “polymeric compound” OR polyethylene OR polypropylene OR “polymer modifier” OR “polymer additive” OR “plastic waste”) AND (“reclaimed aggregate” OR “recycled aggregate” OR RAP OR “reclaimed asphalt pavement” OR “construction and demolition waste” OR C&DW)) AND DT = (Article OR Review))”
580	234		301
Total		1115	

**Table 6:** Significant studies compliant with the goals of the review.

Refs.	Materials	Tests and Methods	Key Takeaways	Gaps or Limitations
[9]	Plastomers Elastomers Rubber	Dynamic Shear Rheometer Viscosity Elastic recovery Multiple Stress Creep and Recovery PG Grading Creep	Enhanced rutting resistance, improved fatigue resistance, increased high temperature stiffness, and reduced cracking. Plastomers improve high temperature stiffness but may be brittle Elastomers enhances resistance to rutting and fatigue Rubber improves resistance to fatigue but has dispersion issues.	Lack of long-term performance data and issues with rubber dispersion.
[11]	SBS, SBR, EVA, Rubber	Rheological Analysis Spectroscopy Microscopy XRD	SBS improves rutting and aging resistance, SBR enhances elasticity, and wax enhances workability in the mixture.	Limited focus with SBS on high temperature stability.
[45]	Recycled plastics (dry/wet process), SBS, EVA, PE, PP, PET	wheel tracker tests stiffness rutting fatigue	Enhanced stiffness, resistance fatigue, rutting, superior moisture recycled plastic modified asphalts. Recycled plastic enhances rutting and fatigue resistance.	The need for further research on the performance of Superpave with high recycled content.
[5]	SBS, SBR	Dynamic modulus and fatigue resistance	Improved fatigue resistance and moisture susceptibility, better rutting resistance than Marshall. SBS and SBR improve fatigue and rutting resistance.	Lack of long-term field validation and economic feasibility.
[3]	SBS, SBR	Volumetrics Mechanical characteristics of Marshall and Superpave	Improved rutting and fatigue resistance especially in high-temperature fluctuations. Superpave outperforms Marshall under heavy traffic and extreme temperatures.	Limited data on Superpave’s applicability in low-income regions.
[46]	SBS, RAP Virgin Asphalt	Gyratory Compaction Dynamic modulus test Cyclic direct tension test FlexPave^TM^ (version 1.1) software	Enhanced rutting resistance with high RAP content but may reduce flexibility.	Performance effect of high RAP content on low temperature remains underexplored.
[47]	SBR, SBS and Crumb Rubber	Fatigue test and wheel tracking	Enhanced fatigue and aging resistance particularly at high temperatures. SBS and SBR performs enhanced high temperature fatigue resistance while crumb rubber provides better ductility.	Limited studies on long term field performance on crumb rubber modified asphalts.
[48]	Recycled concrete aggregate	Marshall compaction, creep test, tensile strength ratios	Lower deformation resistance with fine graded mixtures, improved rutting resistance in coarse mixtures. Mixtures gradation has bigger impact on rutting resistance than RCA content.	Lack of long-term performance with recycled concrete aggregate (RCA) in asphalt mixtures.
[49]	SBS, PE	tensile strength ratios uniaxial dynamic modulus testing	Enhanced stiffness and moisture susceptibility resistance. SBS enhance flexibility and PE improves high temperature stability.	Need for more studies on EVA modified asphalt in low-temperature areas.
[50]	PE, EVA	Tensile strength dynamic modulus	Improved rutting and cracking resistance, particularly in heavy-traffic and high-temperature areas. PE and EVA enhance high temperature performance; EVA may cause brittleness at low temperatures.	Need for more studies on EVA long term field performance.
[30]	Graphene, recycled plastic	Lab tests, Falling Weight Deflectometer, fatigue and rutting test.	Improved stiffness, fatigue resistance, and rutting performance. Graphene enhances stiffness and fatigue resistance, while recycled plastics reduce carbon footprint.	Need for long-term filed performance data for graphene modified asphalt.
[28]	Recycled plastic	Rheological tests, stiffness and penetration	Multicycle recyclability without loss of performance, stable binder properties, recycled plastics shows high recyclability with stable binder properties across multiple cycles and hence promotes sustainability.	Limited research on environmental impact assessment (EIA), and performance in extreme climates.
[7]	Recycled plastic, EVA	Fatigue test	Recycled plastic offers excellent sustainability and mechanical performance across multiple cycles.	Need for more studies on impact of recycled materials aging.
[51]	RAP, virgin asphalt	Gyratory compaction, fracture toughness test	RAP improves fracture resistance but may decrease shear resistance	Further investigation on RAP’s impact on cracking and shear resistance needed

## Data Availability

No new data were created or analyzed in this study. Data sharing is not applicable to this article.

## Abbreviations

The following abbreviations are used in this manuscript:

PMB	Polymer-Modified Bitumen
PMA	Polymer-Modified Asphalt
SBS	Styrene–Butadiene–Styrene
SBR	Styrene–Butadiene Rubber
SEBS	Styrene–Ethylene–Butylene–Styrene
RAP	Reclaimed Asphalt Pavement
RCA	Recycled Concrete Aggregate
DSR	Dynamic Shear Rheometer
PG	Performance Grade
ITS	Indirect Tensile Strength
ITSR	Indirect Tensile Strength Ratio
EVA	Ethylene-Vinyl Acetate
PE	Polyethylene
LDPE	Low-Density Polyethylene
HDPE	High-Density Polyethylene
XRD	X-ray Diffraction
PHBV	β-hydroxybutyrate-co-β-hydroxy valerate
FTIR	Fourier Transform Infrared Spectroscopy
